# Designing a national model for assessment of nursing informatics competency

**DOI:** 10.1186/s12911-021-01405-0

**Published:** 2021-02-02

**Authors:** Mehrdad Farzandipour, Hashem Mohamadian, Hossein Akbari, Samira Safari, Reihane Sharif

**Affiliations:** 1 Health Information Management Research Center, Department of Health Information Management and Technology, School of Allied Health Professions, Kashan University of Medical Sciences, Pezeshk Blvd, 5th of Qotbe Ravandi Blvd – Pardis Daneshgah, Kashan, Islamic Republic of Iran; 2 Research Centre for Health-Related Social Determinates, Department of Health Education and Promotion, Ahvaz Jundishapur University of Medical Sciences, Ahvaz, Islamic Republic of Iran; 3 Department of Biostatistics and Epidemiology, School of Health, Kashan University of Medical Sciences, Kashan, Islamic Republic of Iran

**Keywords:** Average variance extracted, Composite reliability, Confirmatory factor analysis, Nursing informatics competency, Assessment

## Abstract

**Background:**

Due to the need for informatics competencies in the field of nursing, the present study was conducted to design a psychometric instrument to determine the qualification of informatics competencies of employed nurses in educational care centers.

**Methods:**

The questionnaire was made by reviewing existing scientific resources and assessment tools. Two hundred nurses were selected using simple random sampling. Structural equation modeling was used using the measurement model technique and the average variance was calculated. Linear structural relations (LISREL) software was used to test the assumptions and correlations of the model.

**Results:**

Findings showed relatively good estimation in the fit of first-order measurement model. The informatics knowledge subscale with a determining rate of 0.90 had the greatest explanatory effect among the subscales and informatics skill with a determining rate of 0.67 and basic computer skill with a determining rate of 0.60 were observed. The second-order measurement model of fitness indicators showed that the three factors can well explain the multidimensional construct of informatics competency.

**Conclusions:**

The designed tool can be used to develop educational strategies in relation to nursing students in the field of informatics and prepare them in the rich environment of information technology, which can be helpful in training nursing instructors.

## Background

Health informatics is the interdisciplinary study of the design, development, adoption and application of IT-based innovations in healthcare services delivery, management and planning that covers a wide range of applied subfields including medical informatics, consumer health informatics, Bioinformatics, nursing informatics, dental informatics and public health informatics [1]. Informatics in these terms refers to the ‘‘computer science and study of computational systems that is broader context than information science that encompasses all aspects of the computer environment’’ [2]. Nursing informatics (NI), a subset of health informatics, is an established and growing specialty in the nursing field [3], which is defined as a science and practice that integrates nursing, information, and knowledge with information and communication technologies to enhance the health of people, families, and communities worldwide [4]. The HIMSS Nursing Informatics Workforce Survey (2020) showed that nursing informatics plays an important role in the development, implementation, and optimization of information systems and applications, including clinical documentation (CD), computerized provider order entry (CPOE), and electronic health records (EHR) [5]. There is evidence that awareness of the nursing informatics competencies is necessary to fulfill their professional responsibilities [6].

Competence is defined as the state of being well qualified, and it means having the knowledge, skills, and ability to perform specific tasks, activities, or professions [7]; however, it also includes the concept of values, attitudes, critical thinking, and clinical decision-making [8]. Therefore, nursing informatics competencies can be defined as adequate knowledge, skills, and abilities to perform specific informatics tasks [9]. Today, to maintain public health as one of nursing education's main goals, much emphasis has been placed on evaluating and empowering nursing informatics competencies [10, 11]. Without informatics standards and competencies, information technology would be ineffective in the healthcare setting and impose risks on patient safety.

By 1988, nurses in the International Medical Informatics Association and the National League for Nursing developed the first informatics competencies, and other studies soon followed [12, 13]. However, these identified competencies only described entry-level competencies such as computer skills for nurses, rather than the more sophisticated informatics skills needed by experienced nurses, especially informatics nurse specialists (INS). Renewed interest in informatics competencies for nurses began in the early 2000s. Staggers et al. [14–16] defined the initial standard for determining, ranking, and evaluating nursing informatics competencies. This was the first study to cover four levels of nursing, developed competencies for both entry-level and experienced INS, and examined the categories of computer skills, informatics knowledge, and informatics skills [14].

In Iran, as a developing country, the need to consider the concept of nursing informatics competencies has been emphasized in recent years. Some of the topics related to competency mainly focus on clinical competencies, such as nursing managers, nurse educators, nursing students, and nursing graduates [6]. However, extensive research in this area is limited and so far, only a few studies have been conducted concerning the concept of the nursing informatics competencies in this country. In a study (2019), the effect of a training program was examined on the nursing informatics competencies of critical care nurses in Iran based on the Nursing Informatics Competency Assessment Tool (NICAT) [17]. This tool is developed based on the need for an acute care setting and is not generalizable to all healthcare settings [18]. Therefore, due to the changes in technology and newer technologies required in nursing, more attention should be paid to the informatics competencies for the development and standardization of this concept in various aspects of the nursing profession. To that end, the present study aimed to design a psychometric instrument to qualify the informatics competencies of nurses employed in the educational health care centers.

## Methods

### Study design, setting and participants

This descriptive, cross-sectional study was conducted in Iran in 2018 to investigate the psychometric properties and factor structures of nursing informatics competencies. Eligible nurses with Bachelor's degrees participating in this study needed to have full-time job experience of at least 3 years in eight teaching hospitals in the country. Table 1 shows the research outline in January-October, 2018.

**Table 1:** Research outline

Executive activities	Total time
Collecting data, filling in the questionnaires, and entering the data into the software	(January-April 2018) 4 months
Information analysis	(May–July 2018) 3 months
Reviewing, concluding and writing the manuscript	(August-October 2018) 3 months
Total	10 Months

### Questionnaire development

After a review of previous studies and evaluation of existing tools and scientific resources associated with nursing informatics competencies, the basic items of the questionnaire were extracted and then classified into three domains (computer skills, informatics knowledge, and informatics skills) based on the study conducted by Staggers et al. (2002) [14]. The questionnaire consisted of 74 items at three subscales: Basic Computer Skills (22 items), Informatics Knowledge (25 items), and Informatics Skills (27 items). It should be noted that computer skills in this study is defined as the proficiency in the use of computer hardware and software and should not be confused with computer science.

The questionnaire was reviewed in a focus group of twelve experts, including eight nursing experts holding a Master's degree or faculty members with at least five years of work experience with hospital information systems (HIS) and four information technology (IT) professionals holding a Bachelor's degree with at least five years of work experience in the IT department of the hospital. Experts scored the importance of each item on a 5-point Likert scale ranging from ‘strongly disagree’ to ‘strongly agree’. The average importance of informatics competencies items was estimated separately for each item, and then the final questionnaire was designed.

The content validity of the questionnaire was assessed by the same experts using the content validity ratio (CVR) [19] and content validity index (CVI) [20]. In CVR, experts' responses for each item were measured based on the three scales of (1) 'necessary', (2) 'helpful but not necessary', and (3) 'not necessary'. Then, according to the Lawshe table[21], items with a content validity ratio (CVR) higher than 0.56 were considered acceptable. To calculate CVI, relevance, clarity, and simplicity of all items were checked using a 4-point Likert scale, and items with CVI > 0.79 were considered appropriate. Finally, the items were confirmed in three basic computer skills (17 items), informatics knowledge (15 items), and informatics skills (16 items) subscales. Following validity assessment, content reliability of the questionnaire was assessed using the test–retest method. Thus, a valid version of the questionnaire was administered to 20 nurses. After two weeks, the participating nurses were asked to fill out the questionnaire again. Cronbach's alpha was used to assess data reliability. Data reliability was calculated as 0.97.

### Data collection

Before conducting the research, verbal consent was obtained from all participants. They were informed that they could withdraw from the study at any time during filling out the questionnaires if they do not want to continue. All questionnaires were anonymous, and the participants were assured of the confidentiality of their information. This study was approved by the ethics committee of Kashan University of Medical Sciences (KAUMS).

### Study size

The confirmatory factor analysis (CFA) designed to validate an appropriate and meaningful instrument was used to assess nursing informatics competencies. In this study, the sample comprised 200 nurses eligible for the study. According to Kline [20], in the factor analysis, the minimum sample size of 200 is defensible. Thus, the sample size of the present study was larger because, in the confirmatory factor analysis, the minimum sample size is determined based on the subscales, not the items. All samples were selected using a simple random sampling technique according to a list of nurses with a Bachelor's or higher degree and three years of work experience with Hospital Information System (HIS) in teaching and medical hospitals.

### Data sources and measurement

The questionnaire comprising two parts was distributed among nurses. The first part included demographic characteristics including age, sex, marital status, employment, level of education, work experience, familiarity with computers, frequency of computer usage, and interactions with the HIS. The second part included questions related to the subscale of informatics competencies on a scale of 48 questions.

The validity of the constructed instrument was analyzed through confirmatory factor analysis [22] using LISREL software (version 8.5). LISREL has also been used for the structural equation modeling in two parts: confirmatory factor analysis and path analysis. In order to assess the amount of fitness in the developed measurement model, confirmatory factor analysis and experimental data were used to get the chi-square index, chi-square goodness of fit index, adjusted goodness of fit index, comparative fit index, and the root mean square error of approximation (RMSEA) [23]. In this study, structural equation modeling was used with the help of measurement model, composite reliability, and construct validity (convergent and discriminant) using the maximum likelihood approach through LISREL 8.5 software to test the assumptions and correlations of the model.

Convergent validity shows the compatibility of items measuring the same construct. Three methods were evaluated to achieve convergent validity and solidarity: (a) factor loading suggested by Haire et al. [24] as the accreditation criterion for the factor loading of the numeric amount of 6.0 and above. It is noteworthy that if the amount of the factor loading of an item in conjunction with a construct is higher, the item plays a greater role in explaining the constructs. If the factor loading is less than 3.0, the role of the item is not significant, so that it will be ignored. The factor loading between 3.0 and 6.0 is considered acceptable. If it is greater than 6.0, it will be desirable. (b) Composite reliability shows the number of item reflections on the desired construct. The amount of the proposed criterion of composite reliability is 7.0, confirming that the reliability is acceptable [25]. (c) The average extracted variance is equal to or more than 0.5 [26, 27]. In discriminant validity, the amount of difference between the items of the construct and those of other constructs was evaluated. The most common way to assess discriminant validity needs to be formed in the correlation matrix (Fornell and Larker) [28]. Therefore, the original diagonal values are the root of Average Variance Extracted (AVE) coefficients of each construct, and lower values of original diagonal are the correlation coefficients of each construct with other constructs. Based on the accreditation criterion, the original diameter values should be greater than the diameter.

## Results

### Demographic characteristics

Out of 200 questionnaires distributed, 197 were completed. The response rate was 98.5%. About 77% of the respondents were employed in the position of a ward nurse, and 11.5% in the position of a head nurse. The majority of participants were females (75.5%) and married (68.5%). Regarding the level of education, 95.6% had a Bachelor's degree with 3 to 11 years of work experience (56.3%). In terms of familiarity with computers, 66.5% were relatively good, 59% of nurses used computers more than once a day, and spent less than 1 h working with the HIS in each shift (59%).

### Correlation between Demographic characteristics and nursing informatics competencies

The correlation analysis results revealed no significant relationship between sex, marital status, level of education, work experience, frequency of computer usage, and interaction with the HIS and nurses' basic computer skills (P > 0.05). However, there was a significant relationship between age, familiarity with computers, and nurses' basic computer skills (P < 0.02). The results did not show any significant relationship between sex, age, level of education, work experience, and frequency of computer usage, and nursing informatics knowledge (P > 0.05). Nevertheless, there was a significant relationship between marital status, familiarity with computers, and interaction with the HIS and nursing informatics knowledge (P < 0.02). In the subscale of informatics skills, there was no significant relationship between sex, age, marital status, level of education, work experience, and frequency of computer usage and nursing informatics skills (P < 0.05), while a significant relationship was observed between familiarity with computers, interaction with the HIS and nursing informatics skills (P < 0.02).

### Validity of the questionnaire

Table 2 shows CVI and CVR for each item. Among the questionnaire items, five items from the basic computer skills, 10 items from informatics knowledge, and 11 items from the informatics skills subscales were removed. Therefore, the number of items in the questionnaire was reduced to 48 (Additional file 1).

**Table 2:** Validity in quantitative content analysis

Row	Basic computer skills	CVI			CVR	Result
		Simplicity	Clarity	Relevancy		
1	Having basic computer skills (e.g. turning on and turning off the computer, printing, documentation, using the mouse)	0.75	0.75	0.83	1	Confirmed
2	Using windows operating system	0.91	0.91	1	0.8	Confirmed
3	Being able to resolve common error reports	0.75	0.7	0.75	0.8	Confirmed
4	Using an antivirus software to scan files, folders and drives	1	0.9	0.7	0.6	Confirmed
5	Being able to back up computer files	0.91	0.9	0.75	0.6	Confirmed
6	Operating peripheral devices such as printers and scanners	0.83	0.83	0.75	0.6	Confirmed
7	Using spreadsheet applications such as Microsoft excel	0.83	0.91	0.91	0.6	Confirmed
8	Using external storage devices such as CDs, DVDs and memory cards	0.83	0.7	0.75	0.6	Confirmed
9	Changing the default printer from the installed printer list	0.83	0.83	0.91	0.6	Confirmed
10	Using multimedia presentation devices	0.33	0.25	0.41	0.33	Rejected
11	Using word processing software applications	0.91	0.91	1	0.8	Confirmed
12	Having typing skills	1	0.91	0.91	0.6	Confirmed
13	Using PowerPoint software application	1	0.91	0.91	0.56	Confirmed
14	Using Access software application	0.91	0.91	0.58	− 0.5	Rejected
15	Using the Internet	0.91	0.91	0.83	0.77	Confirmed
16	Using search engines on the Internet	1	1	0.91	0.8	Confirmed
17	Using e-mails (e.g. sending mails, responding to mails, attaching files, forwarding mails and deleting mails)	0.91	1	0.91	0.6	Confirmed
18	Using the computer safely	0.75	0.7	0.7	1	Confirmed
19	Using computerized self-learning equipment	0.91	0.91	0.91	0.8	Confirmed
20	Installing windows operating system	0.83	0.83	0.33	− 0.4	Rejected
21	Using telecommunication devices such as modems or other devices to communicate with other systems	0.83	0.83	0.75	− 0.2	Rejected
22	Using the techniques of encryption and access control	0.58	0.41	0.36	− 0.33	Rejected

	Informatics knowledge	CVI			CVR	Result
		Simplicity	Clarity	Relevancy		
23	Knowing the common computer terminology, e.g., bit, byte, RAM, ROM, etc	0.91	0.91	0.58	− 0.2	Rejected
24	Knowing basic components of a computer's hardware system and their functions	0.75	0.75	0.72	0.2	Rejected
25	Knowing the usage of file management function in computer operating system	0.83	0.83	0.7	0.56	Confirmed
26	Knowing how to install software drivers for accessories	0.75	0.75	0.41	− 0.2	Rejected
27	Describing information needed through key concepts and terms in nursing profession	0.75	0.75	0.7	0.56	Confirmed
28	Determining the most appropriate methods for accessing information electronically	0.72	0.75	0.7	0.6	Confirmed
29	Evaluating health information on the Internet using a structure critique format	0.41	0.54	0.63	0.2	Rejected
30	Recognizing that there are human functions that cannot be performed by computers	0.7	0.75	0.7	0.6	Confirmed
31	Knowing the probability of making mistakes by computer users	0.81	0.75	0.81	0.6	Confirmed
32	Knowing the importance of confidentiality and privacy when processing computerized data and medical records	0.72	0.75	0.9	0.6	Confirmed
33	Applying the principles of data integrity, professional ethics and legal requirements for patient confidentiality and data security	0.72	0.72	0.91	0.6	Confirmed
34	Understanding the essentials of information sources such as a variable form, different characteristics, and various physical formats	0.3	0.4	0.6	− 0.14	Rejected
35	Understanding and applying essential information-seeking concepts and practices	0.83	0.7	0.75	0.6	Confirmed
36	Understanding the procedure of scholarly information	0.66	0.58	0.83	0.2	Rejected
37	Analyzing patient information needs, accesses technology resources etc. to meet the needs and to evaluate effectiveness	0.75	0.75	0.81	0.56	Confirmed
38	Recognizing the need for continual learning informatics skills, applications, and knowledge	0.83	0.83	0.91	0.6	Confirmed
39	Recognizing that a computer program has limitations due to its design and computer capacity	0.75	0.75	0.7	0.6	Confirmed
40	Recognizing that it takes time, persistent effort, and skill for computers to become effective tools	0.91	0.83	0.91	0.6	Confirmed
41	Knowing about the laws regarding protecting personal information in computers	0.83	0.91	0.91	1	Confirmed
42	Recognizes that one does not have to be a computer programmer to use computers in nursing effectively	0.83	0.91	0.83	0	Rejected
43	Recognizing that computers are not intelligent in themselves and must be programmed based on users’ needs	1	1	0.83	− 0.2	Rejected
44	Describing patients’ rights as pertaining to computerized information management	0.54	0.41	0.9	− 0.6	Rejected
45	Recognizing the use and importance of nursing data for improving practice	0.91	0.75	0.91	1	Confirmed
46	Recognizing when information and communicates are needed	0.83	0.75	0.75	0.56	Confirmed
47	Recognizing the value of clinicians’ involvement in the design, selection, implementation, and evaluation of applications and systems in healthcare	0.66	0.66	0.66	0	Rejected

	Informatics skills	CVI			CVR	Result
		Simplicity	Clarity	Relevancy		
48	Capturing data and information related to clinical care	0.7	0.91	0.91	0.6	Confirmed
49	Using wireless devices to locate and download resources for patient safety and quality care	0.66	0.75	0.58	0.4	Rejected
50	Using the HIS for nursing records	1	1	1	0.8	Confirmed
51	Using the HIS to store, retrieve and transfer patient data	1	1	1	0.77	Confirmed
52	Using applications for nursing diagnostic coding	0.91	0.81	0.72	0.6	Confirmed
53	Extracting data from clinical data sets	0.7	0.75	0.81	0.8	Confirmed
54	Accessing shared data sets	0.75	0.7	0.9	0.6	Confirmed
55	Participates in influencing the attitudes of other nurses toward computer use for nursing practice	0.83	0.83	0.77	0.56	Confirmed
56	Using wireless devices to locate and download resources	0.36	0.45	0.54	− 0.2	Rejected
57	Accessing, entering and retrieves local data for patient care (e.g. using the HIS and CIS for plans of care, assessment, intervention, notes and discharge planning	0.75	0.75	0.9	0.6	Confirmed
58	Using database management programs to develop a simple databases or tables	0.66	0.58	0.63	0	Rejected
59	Using an application to plan patient care including discharge planning	0.83	0.9	0.83	0.6	Confirmed
60	Using database software programs to construct nursing databases	0.66	0.72	0.33	0.2	Rejected
61	Assessing health information accuracy online	0.54	0.63	0.72	0.2	Rejected
62	Applying patient tele-monitoring systems	0.91	0.75	0.75	0.11	Rejected
63	Selecting system or application software programs	0.66	0.5	0.54	0.33	Rejected
64	Participating in selection, design, implementation and evaluation of systems	0.83	0.75	0.7	0.56	Confirmed
65	Teaching users and clients of nursing systems	0.83	0.7	0.77	0.6	Confirmed
66	Correcting some of the defects observed in working with systems	0.83	0.91	0.7	0.8	Confirmed
67	Applying information management technology for patient education	0.91	1	0.91	0.8	Confirmed
68	Using related social networks on the Internet	0.91	0.72	0.58	0	Rejected
69	Using multimedia files for learning	0.75	0.91	0.83	0.56	Confirmed
70	Being capable of creating and managing blogs or websites	0.91	0.91	0.58	− 0.11	Rejected
71	Using Endnote software application	0.91	0.83	0.5	0	Rejected
72	Using statistical software programs such as SPSS and other software programs for nursing information analysis	0.91	1	0.58	0.2	Rejected
73	Using library information and downloading nursing articles	1	1	0.75	0.8	Confirmed
74	Using search engines	1	0.91	0.83	0.8	Confirmed

CVI: Content Validity Index; CVR: Content Validity Ratio; CD: Compact Disc; DVD: Digital Versatile Disc; RAM: Random Access Memory; ROM: Read-Only Memory; HIS: Hospital Information System; CIS: Clinical Information System; SPSS: Statistical Package for the Social Sciences

### Confirmatory factor analysis of the Questionnaire

**Fig. 1:**
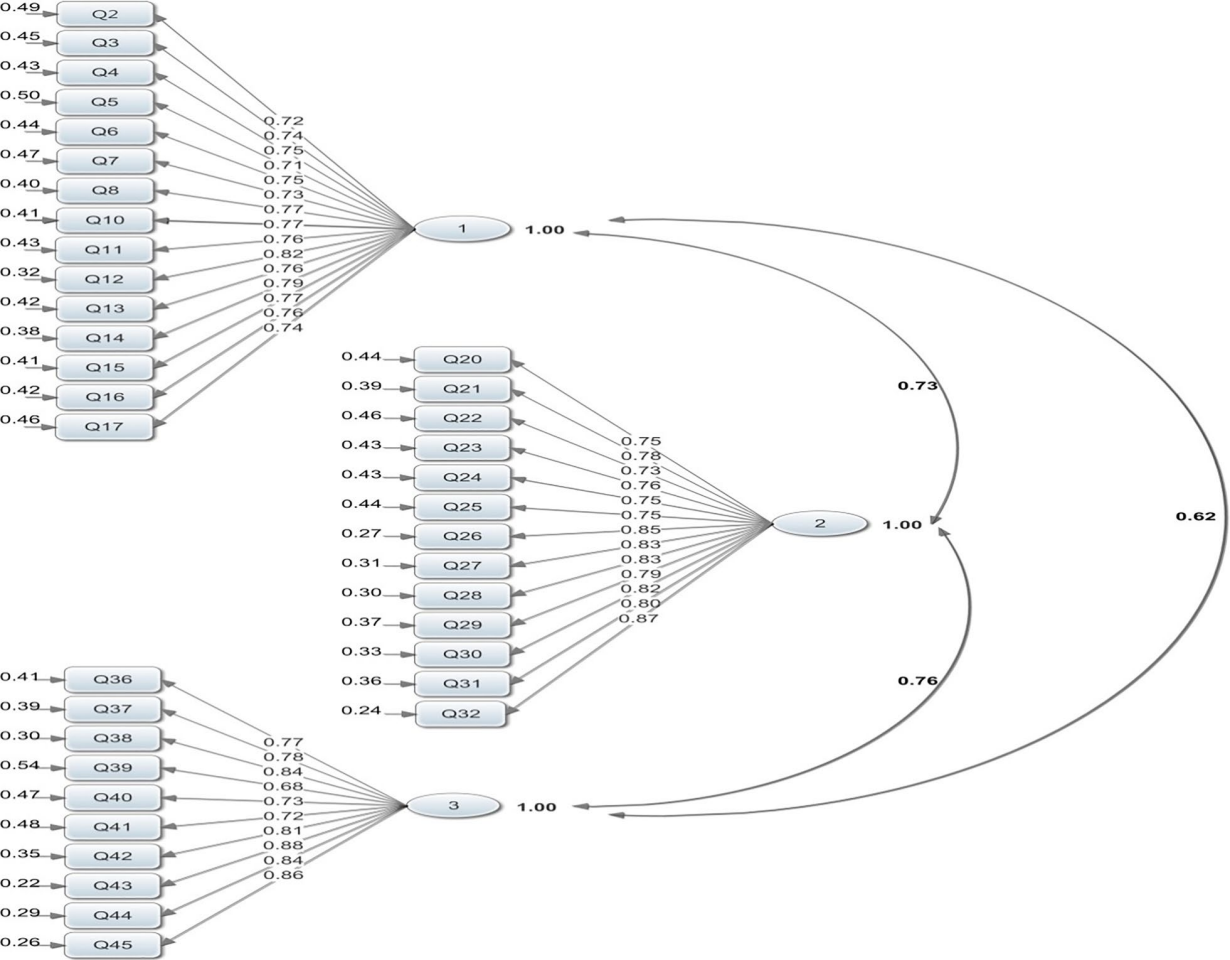
The three-factor structure of first order confirmatory factor analysis of nursing informatics competency**.** Chi-square = 983.61, df = 616, p-value = 0.00001, RMSEA = 0.055

To determine which item belongs to each factor, the confirmatory factor analysis was used. The results showed a relatively good estimation in the fit of the first-order measurement model (χ^2^/df = 1.59, GFI = 0.79, CFI = 0.99, NFI = 0.97, RMSEA = 0.055). It is worth mentioning that each subscale was separately analyzed through the confirmatory factor analysis using the LISREL, such that some items were removed to obtain a relatively appropriate measurement model (Fig. 1). Two items of basic computer skills and informatics knowledge and six items of informatics skills subscales were removed because the cut-off point was lower than 0.5 (R^2^ < 0.5).

**Fig. 2:**
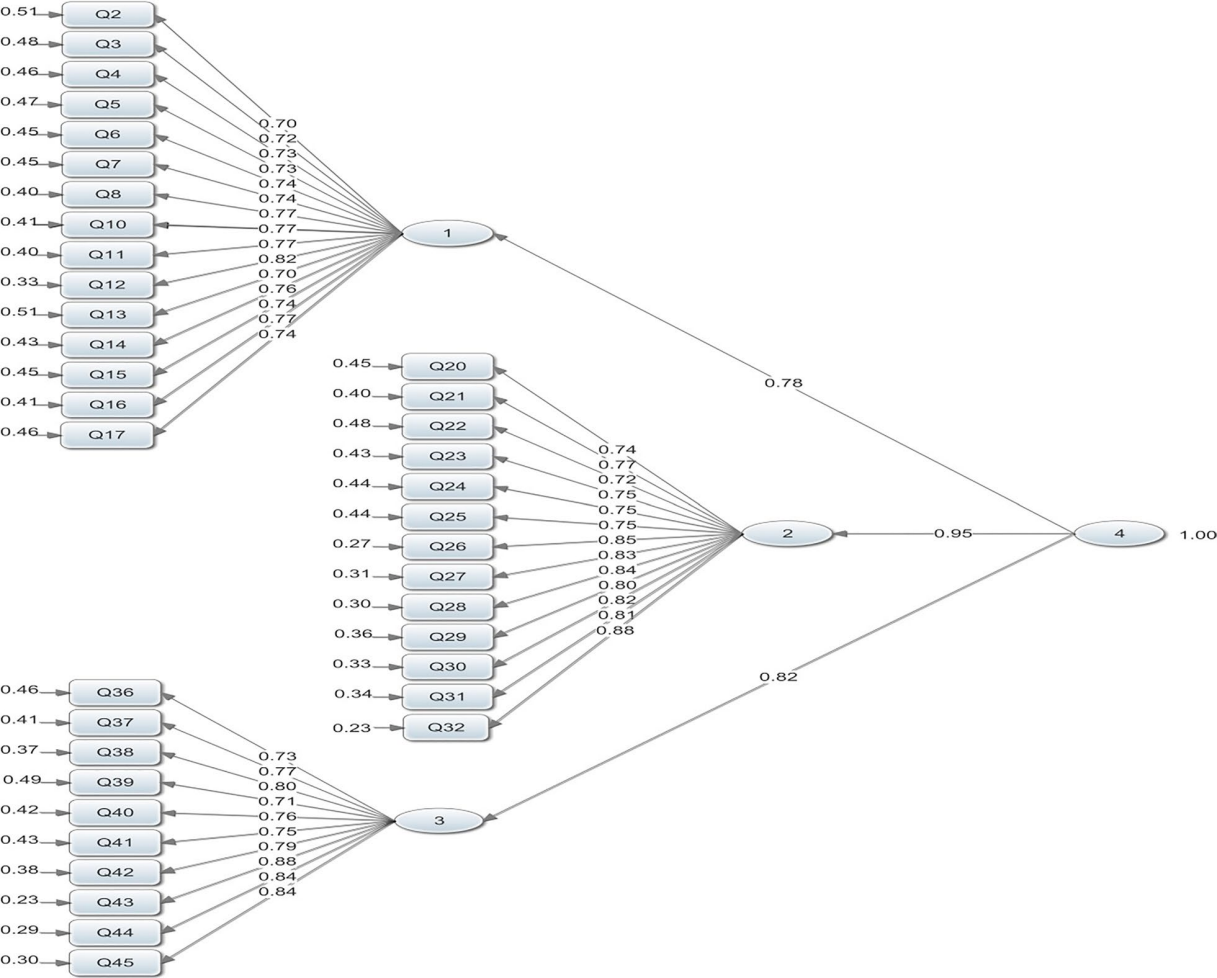
The three-factor structure of second order confirmatory factor analysis of nursing informatics competency**.** Chi-square = 931.57, df = 613, p-value = 0.00001, RMSEA = 0.051

Table 3 shows the discriminant validity and reliability for each subscale. Furthermore, to examine the fit of subscales, the subscales were analyzed along with all items in the first-order measurement model. Therefore, none of the items were removed (Fig. 1). Finally, the second-order factor analysis was used to evaluate the effect of basic computer skills, informatics knowledge, and informatics skills on nursing informatics competencies and assess whether the entire subclass was put into the form as a concept (Fig. 2). It was observed that the subscale of informatics knowledge with the determining rate of 0.90 has the greatest explanatory effect among the subscales and informatics skills with the determining rate of 0.67 and basic computer skills with the determining rate of 0.60 were ranked second and third.

**Table 3:** Reliability and construct validity

Items	Subscale	Factor loading	R^2^	T-value	CR	AVE
	*Basic computer skills*				*0.56*	*0.95*
1	Using windows operating system	0.73	0.53	11.56		
2	Being able to resolve common error reports on the computer	0.76	0.57	12.21		
3	Using the antivirus software program to scan files, folders and drives	0.76	0.58	12.34		
4	Being capable of backing up computer files	0.73	0.53	11.63		
5	Operating computer accessories such as printers and scanners	0.76	0.58	12.30		
6	Using spreadsheet applications such as Microsoft Excel	0.75	0.56	12		
7	Using external storage devices such as CD-ROMs, DVD ROMs, memory disks etc	0.79	0.63	13.11		
8	Using word processing software programs	0.74	0.55	11.90		
9	Having typing skills	0.73	0.54	11.71		
10	Using PowerPoint software application	0.81	0.65	13.54		
11	Using the Internet	0.70	0.50	10.95		
12	Using search engines on the Internet	0.76	0.57	12.22		
13	Using e-mails (e.g. sending mails, responding to mails, attaching files, forwarding mails and deleting mails)	0.75	0.56	11.98		
14	Using computer-based technologies safely	0.77	0.60	12.62		
15	Using computerized self-learning equipment	0.70	0.50	10.91		
	*Informatics knowledge*				*0.95*	*0.65*
16	Determining the most appropriate methods for accessing information electronically	0.71	0.51	11.37		
17	Recognizing that there are human functions that cannot be performed by computers	0.78	0.61	12.89		
18	Knowing the probability of making mistakes by computers	0.73	0.54	11.78		
19	Knowing the importance of confidentiality and privacy when processing computerized data and medical records	0.76	0.57	12.28		
20	Applying the principles of data integrity, professional ethics and legal requirements for patient confidentiality and data security	0.77	0.60	12.61		
21	Understanding and applying essential information-seeking concepts and practices	0.74	0.54	11.83		
22	Analyzing patient information needs and accessing technology resources to meet needs and evaluate effectiveness	0.79	0.63	13.11		
23	Recognizing the need for continual learning of informatics skills, applications, and knowledge	0.85	0.72	14.62		
24	Recognizing that a computer program has limitations due to its design and computer capacity	0.82	0.68	13.97		
25	Recognizing that it takes time, persistent effort, and skill for computers to become effective tools	0.81	0.65	13.55		
26	Knowing about the laws regarding protecting personal information in computers	0.81	0.66	13.63		
27	Recognizing the use and importance of nursing data for improving practice	0.83	0.69	14.05		
28	Recognizing when information and communicates are needed	0.88	0.77	15.43		
	*Informatics skills*				*0.94*	*0.61*
29	Using applications for diagnostic coding	0.71	0.50	11.10		
30	Extracting data from clinical data sets	0.75	0.57	12.07		
31	Accesses shared data sets	0.78	0.61	12.59		
32	Participates in influencing the attitudes of other nurses toward computer use for nursing practice	0.71	0.50	11.10		
33	Accessing, entering and retrieves local data for patient care (e.g. using the HIS and CIS for plans of care, assessment, intervention, notes and discharge planning	0.76	0.58	12.38		
34	Using an application to plan care for patient including discharge planning	0.77	0.59	12.52		
35	Participating in selection, design, implementation and evaluation of systems	0.81	0.65	13.27		
36	Teaching users and clients of nursing systems	0.87	0.76	15.15		
37	Correcting some of the defects observed in working with systems	0.85	0.73	14.46		
38	Applying information management technology for patient education	0.84	0.70	14.09		

CR: Composite Reliability; R^2^: R-Squared; AVE: Average Variance Extracted; DVD: Digital Versatile Disc; ROM: Read-Only Memory; HIS: Hospital Information System; CIS: Clinical Information System;

The second-order measurement model of fit indices showed that the three factors of basic computer skills, informatics knowledge, and informatics skills could explain the multidimensional construct of informatics competency and represent the dimensional accuracy in introducing a three-dimensional framework of informatics competency in the best way (χ^2^/df = 1.51, GFI = 0.80, CFI = 0.99, NFI = 0.97, RMSEA = 0.051). Finally, the reliability assessment results from the test–retest method, using Spearman's rank correlation-coefficient (ρ), showed that the nursing informatics competencies tool has the highest correlation in each subscale (more than 0.9).

## Discussion

In the first part of this study, three areas of informatics competency were used as stated by Staggers et al. [14]. Content validity (CVI and CVR) of informatics competency items was assessed by experts with a 48-item validated questionnaire. Staggers et al. [14] designed the classification of 3-factor nursing informatics competencies with 37 items for beginner nurses and 32 items for experienced nurses including basic computer skills, informatics knowledge, and informatics skills. However, evaluation of the need for education at each specific level of nursing informatics competence was not possible [29].

Hunter et al. [9] made the list of nursing informatics competencies in three subscales including basic computer skills (51 items), information literacy (25 items), and clinical data management (9 items). In their study, while basic computer skills were listed in detail, items such as PowerPoint familiarity, Excel familiarity, working with accessories, and typing skills were not listed. In conclusion, it seems that the designed tools in the present study are more complete and more comprehensive in the field of basic computer skills and all the necessary dimensions in this context have been taken into consideration.

Hart [30] performed a three-round Delphi study to determine core informatics competencies for generic nurse managers resulting in a list of 49 core informatics competencies that were categorized into three groups including computer skills, informatics knowledge, and informatics skills. Nonetheless, the list does not mention the practical, research, and educational competencies [29]. Chang et al. [31] conducted a Delphi technique to identify informatics competencies required for nurses in Taiwan. The results showed that 318 informatics competencies for nurses had 97.8% consensus with the results of the study conducted by Staggers et al. [14].

Hwang et al. [32] designed a specific classification for nursing informatics competencies in their study. They classified parts of basic computer skills in the form of informatics knowledge and its original classification; they introduced computer trends structure as one of the three factors affecting nursing informatics competencies in Taiwan whose significance was more than basic computer skills. The difference between the items of these two studies is due to the variety in the classification of nursing informatics competencies structures. Also, the knowledge of using the HIS as an important variable is considered in the informatics knowledge structure in the study conducted by Hwang et al. [32]. This variable was not considered in the present study since it is only the skill of using it that has been classified in the informatics skill structure. This research is the only study assessing nursing informatics competence confirming the three-factor structure including basic computer skills, informatics knowledge, and informatics skills with regard to the suitability of various indicators of structural equation modeling.

Westra et al. [33] introduced 92 items related to nursing informatics competencies based on the work of Staggers et al. [14], including 24 items related to computer skills, 40 items related to informatics knowledge, and 28 items related to informatics skills. This list is the same as the one provided by Hart [30] in providing specific qualifications of access to data, communication, systems, data dissemination, and training. This list is useful for determining individuals’ specific competence while it does not allow the evaluation of the level of competence at a particular level of practice. Westra's list emphasizes the knowledge and skills of informatics in the role of nurses' leadership for planning, supporting, directing, and evaluating information initiatives beyond the practical skills of nurses for action [29].

Lenburg [34] provided an 8-core practice list of essential competencies for nursing including assessment and intervention skills, communication, critical thinking, human caring, teaching, management, leadership, and knowledge integration skills. In competence of communication skills, the sub-skill of computing, it is discussed in which only customer communication, search for resources, and specialized responsibility were provided. In this list, informatics was not presented as a core competence of nursing whereas it is of great importance because of the integration of technology and information communication in contemporary nursing. The difference between Lenburg's study and the present one is that, in the present study, informatics competencies emphasize computer skills and informatics skills when discussing nursing practices.

In another study administered by Cronenwett et al. [35], a list of recommended qualifications was required for all nurses to provide safe care to patients. In the classification provided, at the level of informatics competencies, only 3 subjects including knowledge, skill, and attitude were mentioned. The list is generally a useful tool for identifying current nurses' competencies; however, the informatics section is inadequate in identifying vital concepts of nursing competencies. This study lists the qualifications while it does not show the depth of the skills and the level of training required at each particular level [29].

TIGER research [36] presented a list of essential informatics competencies for experienced nurses including levels of basic computer competence, information literacy, and information management. At the level of basic computer competence, varied items concerning hardware and software intelligence, various forms of electronic communication, recognition of safety and operating systems, using the Internet, and many other items were listed. There are also several competencies in the information literacy and information management area which cannot be evaluated using this list [29]. Integrated informatics was considered to improve education, provide care, and practical measures for nurses [37]. TIGER’s tool was developed for all nurses without specification for level of practice and included 281 competencies. Nevertheless, the use of information technology in the field of health in this country is at an early stage and nursing experts have not presently considered many of the competencies as important and in practice, there is no need to teach them to all nurses. Therefore, nursing informatics can be created in countries that do not have this field, and higher-level activities in the field of clinical health information systems can be expected from them.

In the study by Hubner et al. [38], a broad list of core competencies for nurses was identified based on five nursing domains including clinical nursing, quality management, inter-professional coordination, nursing management, and information technology management. They provided six core informatics competencies in each of the five nursing areas. However, this tool was not carefully developed as the tool demonstrates core competencies, such as nurses’ documentation and information knowledge management, without identifying the competency items under each core competency [39].

Kaminski [40] also introduced a framework determining a specific classification of nursing skills. This list includes a set of competencies required for different levels of nursing. The tool is capable of assessing the presence or absence of nursing skills while it does not show the degree of nursing mastery to the details of the aforementioned skills [29]. In the instrument presented in this study, nursing competence was generally evaluated since nurses were not assessed in terms of their levels in the working field.

The differences between the present study and other studies are due to the differences in concept definitions, the type and level of technology used and the kind of competence that is required. Competencies such as the use of decision support systems, database management systems, database software, remote patient monitoring systems, effective care design tools and appropriate technology to collect patient-related data were considered essential for nurses in Stagger's study. Since in many developing countries, hospital information systems and applications in the field of public health, especially in nursing care, are in the early and experimental stages and have not reached the implementation stage, these competencies were not agreed upon and approved by the experts in this study. The competencies agreed in this area are mostly related to the basic and primary principles of working with computers.

However, the results of this study in terms of construct validity and confirmatory factor analysis as assessed through the 3-factor structure of the Persian version of the nursing informatics competencies questionnaire were different from the results reported in other studies [11, 41, 42]. The reasons behind the difference in the number of factors can be linked to different statistical methods such as exploratory functional analysis concerned with the derivation and determination of the relationships between the factors.

One of the findings of the present study is the relationship between the 3-factor model of nursing informatics competencies and their subscales (Figs. 1, 2). The strongest relationship was observed between informatics knowledge (0.95) and informatics competencies while the weakest relationship was observed between basic computer skills (0.78) and informatics qualifications. In analyzing these results, it is noteworthy to mention that informatics knowledge had a stronger impact on informatics competencies than the other two subscales. Moreover, enhancing nursing informatics knowledge plays a greater role in increasing informatics competencies. In the study conducted by Yang et al. [11], sufficient knowledge regarding technology use significantly increased nurses informatics competencies.

Nevertheless, in a study conducted by Arzu Akman et al. [41], the studied participants had no knowledge of nursing informatics. However, 2.86% of them stated that nursing informatics courses must be included in the nursing curriculum. The computer-based nursing curricula should be dynamically based on the regional needs and adaptations to new professional attitudes to assure the possibility of training the nurses in order to guarantee an adequate level of expertise concerning informatics. The result of this study showed that the questionnaire was reliable, with the minimum reliability coefficient being related to test–retest of informatics skills subscale (0.95) and the highest reliability coefficient being related to basic computer skills subscales (0.99). In the study done by Akman et al. [41], the reliability of the designed tools was proper. The results of the present study showed that participants’ level of informatics capabilities was medium. In other studies, nursing informatics skills are have been shown to be at an intermediate level [11, 42, 43].

### Implications for practice and future research

The presented tool can be used to develop educational strategies for nursing students in the field of informatics and to prepare them in the rich environment of information technology in the healthcare environment of developing countries. Therefore, the Faculty members of Nursing and Midwifery and the Education Deputy of Ministry of Health, Treatment, and Medical Education can benefit from the results of this study. Future nurses cannot be well-prepared to confront challenges in the various professional fields without the acquisition of technical skills. Therefore, nurses should be able to use data, information, knowledge, and technologies properly to improve nursing care. In this regard, nursing informatics can play a significant role in the areas of education, healthcare provision, research, and management and be used for preservation and storage of clinical care and the extension of research studies that are directly related to the quality of patient healthcare.

## Conclusion

Since nurses are regarded as the largest part of the healthcare workforce and the major users of clinical information systems, nursing informatics competencies are important for the successful use of clinical information systems and improving patient safety in computerized environments. Therefore, the nursing profession needs to promote the acceptance and usage of information technology to empower nursing informatics competencies. For this purpose, the informatics competencies approved in the present questionnaire can be helpful for educational administrators in order to train nurses and include a special course for nursing informatics in the educational curriculum. This questionnaire can also be useful in periodically evaluating the level of informatics competencies of working nurses and nursing students in different communities.

## Supplementary Information

**Additional file 1.** English Questionnaire.

## Data Availability

The datasets used and/or analysed during the current study available from the corresponding author on reasonable request.

## Abbreviations

LISREL: Linear structural relations
ICT: Information and Communications Technology
EHR: Electronic Health Records
HIS: Hospital Information System
CVI: Content Validity Index
CVR: Content Validity Ratio
AVE: Average Variance Extracted
RMSEA: Root Mean Square Error of Approximation
CFA: Confirmatory Factor Analysis
GFI: Goodness of Fit Index
NFI: Normed fit index
