# Supplementary material for: Designing a national model for assessment of nursing informatics competency
Source: BMC Med Inform Decis Mak. 2021 Feb 2;21:35. doi: 10.1186/s12911-021-01405-0 (PMC7852364; doi:10.1186/s12911-021-01405-0)
Supplement: Supplementary file 1 — Additional file 1. English Questionnaire. [file 12911_2021_1405_MOESM1_ESM.docx]

**Additional file 1: English Questionnaire**

**Section 1 (General Questions)**

| Job position: ………………....... |
| --- |
| Gender:  Female  Male |
| Age: ……………………… (Year) |
| Marital status:  Single  Married |
| Level of education:  Associate's degree  Bachelor's degree  Masters' degree  Ph.D. |
| Work experience: …………….… (Year) |
| Familiarity with computers:  Very much  Much  Medium  Low  Very low |
| Frequency of using computers:  Several times a day  Once a day  Several times a week  Several times a month |
| Interactions with the HIS in each shift:  Less than 1 hour  Between one and two hours |
| More than two hours |

**Section 2 (Specialized Questions)**

| **Basic computer skills** | **Completely** | **Very much** | **Low** | **Very Low** |
| --- | --- | --- | --- | --- |
| 1. I have basic computer skills (for example, I can turn on and off the computer) |  |  |  |  |
| 2. I have the ability to use the Windows operating system (file management) |  |  |  |  |
| 3. I can solve common errors on my computer |  |  |  |  |
| 4. I have the ability to use the anti-virus software |  |  |  |  |
| 5. I have the ability to backup computer files |  |  |  |  |
| 6. I am able to work with peripheral devices (e.g. scanners, printers) |  |  |  |  |
| 7. I have the skills to use Excel application |  |  |  |  |
| 8. I have the ability to use external storage devices (e.g. CD-ROM) |  |  |  |  |
| 9. I am able to change the default printer from the list of installed printers |  |  |  |  |
| 10. I have the skills to use Word application |  |  |  |  |
| 11. I have typing skills |  |  |  |  |
| 12. I have the skills to use PowerPoint application |  |  |  |  |
| 13. I can use the internet |  |  |  |  |
| 14. I have the ability to use Internet-based search tools |  |  |  |  |
| 15. I am able to use emails (e.g. sending mails, responding to mails, attaching files, forwarding mails and deleting mails) |  |  |  |  |
| 16. I have the ability to use computer system safely (in terms of hardware and software) |  |  |  |  |
| 17. I have ability to use computerized self-learning equipment (such as training CDs) |  |  |  |  |
| **Informatics knowledge** | **Completely** | **Very much** | **Low** | **Very Low** |
| 18. I know how to use the file management function in computer operating system |  |  |  |  |
| 19. I am able to describe the information needed for nursing, among other key terms and concepts |  |  |  |  |
| 20. I am able to determine the most appropriate methods for accessing information electronically |  |  |  |  |
| 21. I have the ability to recognize that some human activities cannot be done by computers |  |  |  |  |
| 22. I am able to understand the probability of making mistakes by computer users |  |  |  |  |
| 23. I know the importance of maintaining confidentiality and privacy in recording clinical data when processing computerized data and medical records |  |  |  |  |
| 24. I have the ability to apply the principles of data integrity, professional ethics and legal requirements for confidentiality and security of patient information |  |  |  |  |
| 25. I can understand and search for key information about the concepts of the nursing profession |  |  |  |  |
| 26. I have the ability to analyze patients’ information needs and access technology resources to meet these needs and evaluate their effectiveness |  |  |  |  |
| 27. I recognize the need for continuous learning informatics skills, applications and knowledge |  |  |  |  |
| 28. I recognize that a computer program has limitations due to its design and computer capacity |  |  |  |  |
| 29. I recognize that it takes time, persistent effort, and skill for computers to become effective tools |  |  |  |  |
| 30. I know the laws regarding the protection of personal information on the computer |  |  |  |  |
| 31. I am able to recognize the use and importance of nursing data for improving practice |  |  |  |  |
| 32. I am able to recognize when information and communication are needed |  |  |  |  |
| **Informatics skills** | **Completely** | **Very much** | **Low** | **Very Low** |
| 33. I am able to collect data and information related to clinical care |  |  |  |  |
| 34. I have the ability to use HIS in the nursing profession such as nursing records |  |  |  |  |
| 35. I have the ability to use HIS to store, retrieve and transfer patient data |  |  |  |  |
| 36. I am able to use applications for nursing diagnostic coding |  |  |  |  |
| 37. I have the ability to extract data from the clinical data sets |  |  |  |  |
| 38. I have the ability to access shared data set in HIS |  |  |  |  |
| 39. I am able to participate in influencing the attitudes of other nurses toward computer use for nursing practice |  |  |  |  |
| 40. I have the ability to access, enter and retrieve the data used for patient care (for example, using HIS and CIS for health plans, assessments, interventions, notes and discharge plans) |  |  |  |  |
| 41. I am able to use software programs and applications to implement a plan of care which includes discharge planning |  |  |  |  |
| 42. I have the ability to participate in the selection, design, implementation and evaluation of systems process |  |  |  |  |
| 43. I am able to teach and guide users and clients of nursing systems |  |  |  |  |
| 44. I have the ability to correct some of the defects observed in working with systems |  |  |  |  |
| 45. I have the ability to apply information management technology for patient education |  |  |  |  |
| 46. I am able to use multimedia files (software, multimedia) for learning |  |  |  |  |
| 47. I have the ability to use relational databases, download nursing articles and publications and my favorite subjects |  |  |  |  |
| 48. I am able to use general and specialized search engines |  |  |  |  |
